# Microcomputed tomography analysis of root canal morfology of hypomineralized permanent molars compared with healthy teeth: unveiling hidden anatomical variations

**DOI:** 10.1186/s12903-026-07919-1

**Published:** 2026-02-17

**Authors:** Hazal Karslıoğlu, Didem Sakaryalı Uyar, Mert Ocak, Hakan Hamdi Çelik

**Affiliations:** 1 Department of Dentomaxillofacial Radiology, Faculty of Dentistry, Mersin University, Mersin, Türkiye Turkey; 2 Department of Pediatric Dentistry, Faculty of Dentistry, Yıldırım Beyazıt University, Ankara, Türkiye Turkey; 3 Anatomy, Department of Basic Medical Sciences, Faculty of Dentistry, Ankara University, Ankara, Türkiye Turkey; 4 Anatomy, Department of Basic Medical Sciences, Faculty of Medicine, Hacettepe University, Ankara, Türkiye Turkey

**Keywords:** Molar incisor hypomineralization, Microcomputed tomography, Root canal morphology, Isthmus, Endodontic treatment

## Abstract

**Background:**

Molar–incisor hypomineralization (MIH) is a prevalent developmental enamel defect that frequently affects permanent molars and is associated with increased dentin permeability, hypersensitivity, and a higher risk of pulpal inflammation. These clinical features may necessitate endodontic treatment at an early age; however, information regarding potential variations in root canal morphology in MIH-affected molars remains limited. This study aimed to investigate the root canal morphology of hypomineralized permanent molars and compare it with that of healthy molars via microcomputed tomography (µCT).

**Methods:**

A total of sixty extracted permanent molars were included in this study, comprising thirty hypomineralized teeth and thirty healthy control teeth. All samples were scanned using a SkyScan 1172 µCT system. Root canal configurations were classified according to the Sert and Bayırlı classification system, and isthmus types were assessed according to the system described by Hsu and Kim. The numbers of major and minor apical foramina were recorded. Morphometric analyses included measurements of canal area, cervical width, and cervical thickness. All the data were statistically analyzed via independent t tests, with the level of significance set at *p* < 0.05.

**Results:**

The most common root canal configuration in both groups was Type I, defined as the presence of two canals without noticeable communication. Hypomineralized teeth exhibited a significantly greater number of minor apical foramina in the middle third of the root (*p* = 0.016) as well as a greater total number of minor apical foramina compared with healthy teeth (*p* = 0.047). The cervical width was significantly greater in the hypomineralized molars (*p* = 0.02), whereas no significant differences were observed in the cervical thickness, canal area, or isthmus distribution between the two groups.

**Conclusions:**

µCT analysis demonstrated distinct morphological differences between hypomineralized and healthy permanent molars, particularly regarding the number of minor apical foramina and cervical width. These findings underscore the need to consider potential root canal variations during endodontic assessment and treatment planning in MIH-affected molars.

## Introduction

Molar incisor hypomineralization (MIH), first introduced in 2001, refers to an enamel defect affecting one to four first permanent molars and frequently the permanent incisors; it may also involve the second permanent molars and permanent canines [1]. Clinically, MIH is characterized by well-demarcated opacities ranging in color from white/yellow to brown. In advanced cases, the hypomineralized enamel, characterized by diminished mineral content, increased porosity, and weakened mechanical strength, is highly susceptible to posteruptive breakdown when exposed to masticatory forces [2]. These structural deficiencies not only facilitate bacterial infiltration beneath apparently intact surfaces but also trigger immune responses within the pulp tissue, thereby increasing susceptibility to early carious lesions [3, 4]. According to a systematic review conducted by Americano et al. [4], children with MIH are 2.1–4.6 times more likely to develop caries in their permanent dentition than are those without MIH.

From a clinical perspective, hypomineralized molars often pose significant restorative challenges. Adhesive restorations in MIH-affected teeth have demonstrated relatively high failure rates, likely due to the increased organic content and altered microstructure of the enamel [5, 6]. Recurrent restoration failure, combined with increased caries susceptibility and posteruptive enamel breakdown, frequently results in irreversible pulpitis at an early age, making endodontic treatment unavoidable in a substantial number of cases [7]. Despite this clinical reality, the potential influence of MIH on root canal morphology—and its implications for endodontic procedures such as canal debridement, disinfection, and obturation—remains insufficiently explored. Morphological complexities, including accessory canals, apical foramina variations, and cervical root canal dimensions, may adversely affect cleaning efficacy and sealing ability, thereby increasing the risk of treatment failure.

Recent microcomputed tomography studies have begun to suggest that MIH may not be limited to enamel alterations alone. A study conducted by Uyar et al. [8] reported that, compared with healthy teeth, hypomineralized teeth presented lower dentin mineral density measurements and a greater number of accessory canals in the middle and cervical regions. These findings indicate that MIH may be associated with deeper structural changes extending into dentin and the root canal system, underscoring the need for a detailed three-dimensional assessment of root canal morphology in affected molars.

Conventional in vitro methods used to evaluate the morphological features of root canal systems often cause irreversible alterations to the tooth, such as sectioning, clearing, and staining. In contrast, microcomputed tomography allows for detailed three-dimensional assessment of root canal morphology without causing any damage to the tooth. Microcomputed tomography has been shown to provide a reliable high-resolution method for detailed morphological assessment. It has enabled the identification of features such as root canal configurations [9–11], canal isthmuses [12], and accessory canals [13, 14].

A thorough understanding of the anatomy of the root canal system is fundamental for the success of endodontic treatment [15, 16]. Nevertheless, despite its clinical importance, there is a paucity of research specifically addressing the root canal morphology of hypomineralized permanent molars.

It was hypothesized that hypomineralized permanent molars would exhibit greater morphological variation in root canal anatomy, including differences in canal configuration, foraminal characteristics, and cervical root canal dimensions, compared with healthy molars.

Therefore, the purpose of this study was to investigate the root canal morphology of hypomineralized permanent molars via µCT. This study evaluated the root canal configuration, cervical width and thickness, root canal areas, prevalence of isthmuses, and number of foramina, with all the parameters being compared between healthy and hypomineralized teeth.

## Materials and methods

According to the results of the power analysis, a minimum of 28 teeth per group—a total of 56 teeth—was determined to be necessary to achieve a statistical power of 90%, with a 5% margin of error and an effect size of 0.8. This study was approved by the Başkent University Institutional Review Board and Ethics Committee [Project No: D-KA22/26].

The study included 60 permanent molars with radiographically confirmed complete root formation, showing no anatomical abnormalities, such as internal resorption, canal obliteration, or root dilaceration, and without any history of previous root canal treatment. Teeth presenting with severe root resorption, incomplete root development, or advanced pathological alterations affecting root morphology were excluded. Among these, 30 were hypomineralized teeth, and 30 were healthy controls. The teeth included in the study were those extracted due to crown destruction, pulpitis, or periodontal and orthodontic reasons.

The age range of the patients from whom the teeth were obtained was 9–16 years. The degree of crown destruction was recorded, and only teeth in which root morphology was not expected to be altered by coronal breakdown were included.

All extracted teeth were collected from patients undergoing dental treatment at the Department of Pediatric Dentistry. Written informed consent was obtained from all patients for the use of their extracted teeth for research purposes, and the study protocol was approved by the institutional ethics committee.

Prior to sample selection, two independent examiners (H.K., D.S.U.) were calibrated to ensure consistency in the diagnosis of molar incisor hypomineralization and in the application of the inclusion and exclusion criteria. Calibration was performed on a subset of extracted teeth that were not included in the final sample. The diagnosis of MIH was carried out based on a surface-based clinical assessment of each tooth. In cases of disagreement between the examiners, a consensus was reached through joint re-evaluation. Teeth for which consensus could not be achieved were excluded from the study.

The inclusion criteria for hypomineralization were based on the clinical signs and symptoms of MIH as defined by Weerheijm et al. and Giuca et al. [1, 17]. Molar teeth exhibiting limited white, yellow, or brown opacities; posteruptive enamel breakdown; extensive restorations; or crown destruction were classified within the hypomineralized group.

The hypomineralized group consisted of molars extracted due to crown destruction, pulpitis, or orthodontic indications from patients who presented with yellow, brown, or white lesions on the buccal surfaces of anterior permanent teeth, ensuring a confirmed diagnosis of hypomineralization.

In contrast, caries-free permanent molars extracted for orthodontic or periodontal reasons and showing no clinical signs of hypomineralization—such as demarcated opacities or posteruptive enamel breakdown—were classified as healthy controls.

All teeth were stored in a chloramine-trihydrate solution at room temperature for a maximum of six months following extraction to prevent dehydration and microbial growth. Prior to µCT evaluation, surface debris was carefully removed, and all samples were sterilized using an autoclave.

µCT evaluation.

The samples [*n* = 60] were scanned via a SkyScan 1172 µCT system [Bruker, Kontich, Belgium]. The X-ray source voltage and current were set at 80 kV and 100 µA, respectively. The samples underwent a full 360-degree rotation in 0.6-degree increments. A 0.5 mm aluminum‒copper filter was used to eliminate the softest X-rays. The resulting image pixel size was 13.68 μm. During scanning, a wet sponge was placed inside the tube to secure the position of the tooth and maintain moisture.

- Root canal configuration: In the present study, the classification of root canal configurations was adopted from the system proposed by Sert and Bayırlı 2004 [18]. This system extends Vertucci’s original classification by incorporating 15 additional configurations that were not previously categorized, providing a more comprehensive framework. Moreover, it has been successfully applied to both maxillary and mandibular molars, making it particularly suitable for assessing the complex root canal anatomy of the teeth included in our study. The choice of this classification system allows for a detailed and standardized evaluation of variations in root canal morphology, which is essential for both clinical and research purposes. The root canals of each root for each tooth were classified.
- Isthmus number and types: The presence and configuration of the isthmuses were evaluated in the cervical, middle, and apical thirds of each root. Isthmus configurations have been classified according to the system described by Hsu and Kim [19]. The Hsu and Kim [19] classification consists of five types:
  - Type I: Presence of two canals without noticeable communication
  - Type II: Presence of two canals with a definite communication
  - Type III: Similar to type II but with three canals instead of two canals
  - Type IV: Extension of the main canal into the isthmus
  - Type V: Presence of a complete communication or corridor between the two canals

- Foraminae: The main apical foramina were defined as openings with a diameter of 0.25 mm or larger, whereas those with smaller diameters were classified as minor foramina. [20, 21] The tooth root was divided into three regions (apical, middle, and cervical), and the number of minor apical foramina in each region was recorded.
- For morphometric analysis, at 1, 3, 5, 7, 9, and 11 mm from the apical foramen, the canal area and cervical width and thickness were measured for each tooth [12, 22]. Cervical width and cervical thickness were selected as morphometric parameters to characterize the coronal portion of the root canal system, as this region is clinically critical for access cavity preparation, coronal flaring, and effective irrigation.

### Statistical analysis

The obtained data were evaluated with the IBM SPSS Statistics V. 22 package program. Descriptive statistics about hypomineralized and healthy teeth were given as numbers. The mean and standard deviation values were given for the root number, major apical foramen, minor apical foramen (cervical, middle, apical, and total), isthmus, cervical thickness, cervical width and areas at 1 mm, 3 mm, 5 mm, 7 mm, 9 mm and 11 mm for the hypomineralized and healthy groups, respectively, as descriptive statistics. Levene’s test was used to evaluate the equality of variances. Independent t tests were used for the analysis of the root number, major apical foramen, minor apical foramen (cervical, middle, apical, and total), isthmus, cervical thickness, cervical width, and areas at 1 mm, 3 mm, 5 mm, 7 mm, 9 mm, and 11 mm. Statistical significance was accepted at *p* < 0.05.

## Results

Sixty permanent molar teeth were divided into 2 groups [*n* = 30]: hypomineralized and healthy. There were 17 maxillary permanent molar teeth and 13 mandibular permanent molar teeth in both groups.

Tables 1 and 2 present the number of roots and the types of root canal configurations of the examined teeth. The most frequently observed root canal configuration was Type I.

**Table 1:** Root Canal configurations of each root of a hypomineralized molar tooth according to Sert and Bayırlı

Hypomineralized teeth (*n*)	Root canal configuration type			Root number
Maxillary molar	MB	DB	*P*	
1	1	1	1	3
2	1	1	1	3
3	1	1	3	3
4	2	1	1	3
5	15	1	1	3
6	1	1	1	3
7	1	1	1	3
8	1	1	1	3

Mandibular molar	Two-rooted		One-rooted	
	M	D		
9			3	1
10	2	1		2
11	4	1		2
12	4	4		2
13			10	1
14	4	1		2
15	4	1		2
16	3	1		2
17			15	1
18	4	1		2
19	4	3		2
20	1	1		2
21			14	1
22	3	1		2
23			3	1
24	2	1		2
25	2	1		2
26			3	1
27	1	1		2
28	1	1		2
29	1	1		2
30	4	1		2

*MB *Mesiobuccal, *DB *Distobuccal, *P *Palatinal, *B *Buccal, *M *Mesial, *D *Distal, n refers to tooth number

**Table 2:** Root Canal configurations of each root of healthy molar teeth according to Sert and Bayırlı

Healthy teeth (*n*)	Root canal configuration type					Root number
Maxillary molar	Three-rooted			Two-rooted		
	MB	DB	*P*	B	*P*	
1	1	1	1	-	-	3
2	3	1	1	-	-	3
3	12	1	3	-	-	3
4	4	10	1	-	-	3
5	4	1	1	-	-	3
6	4	1	5	-	-	3
7	-	-	-	19	1	2
8	-	-	-	10	1	2
9	-	-	-	1	1	2

Mandibular molar	Two-rooted		One-rooted			
	M	D				
10			1			1
11	1	1				2
12	1	1				2
13	3	1				2
14			1			1
15	2	1				2
16	9	1				2
17			1			1
18			1			1
19			15			1
20			6			1
21	4	1				2
22	3	1				2
23	3	1				2
24	4	1				2
25	3	1				2
26			10			1
27			17			1
28	1	1				2
29	4	1				2
30			15			1

*MB *Mesiobuccal, *DB *Distobuccal, *P *Palatinal, *B *Buccal, *M *Mesial, *D *Distal, n refers to tooth number

In the hypomineralized group, maxillary molars predominantly exhibited Type I canal configurations. Specifically, Type I canals were observed in 75% of the mesiobuccal roots, 87.5% of the palatal roots, and 100% of the distobuccal roots. In mandibular molars within the hypomineralized group, Type IV canal configuration was identified in 43.75% of the mesial roots, whereas the distal roots predominantly showed Type I canals (87.5%). In single-rooted mandibular molars of the hypomineralized group, a Type III canal configuration was observed in 50% of the specimens.

In the healthy group, the most frequently observed canal configurations varied according to root type. Among mandibular molars, single-rooted teeth exhibited Type I canal configurations in 44.5% of cases, while in two-rooted mandibular molars, the mesial root most commonly demonstrated a Type III configuration (33%), and the distal root consistently showed a Type I configuration (100%). In healthy maxillary molars, the mesiobuccal root most frequently presented a Type IV canal configuration (50%), whereas the distobuccal and palatal roots predominantly exhibited Type I configurations, observed in 83.3% and 67% of cases, respectively. In two-rooted maxillary molars, the palatal root demonstrated a Type I configuration in all specimens (100%), while the buccal root showed an equal distribution (33.3% each) of Type I, Type 10, and Type 19 canal configurations.

**Fig. 1:**
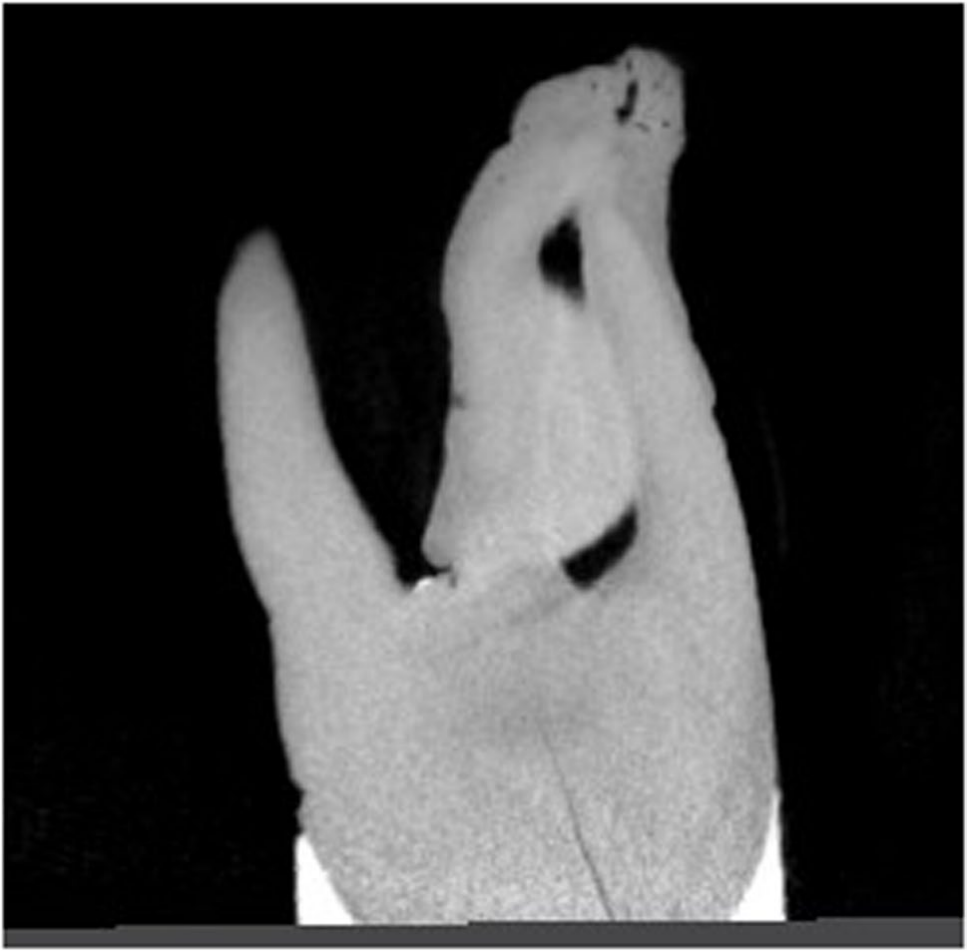
Multiple apical foraminas observed in a healthy tooth

**Fig. 2:**
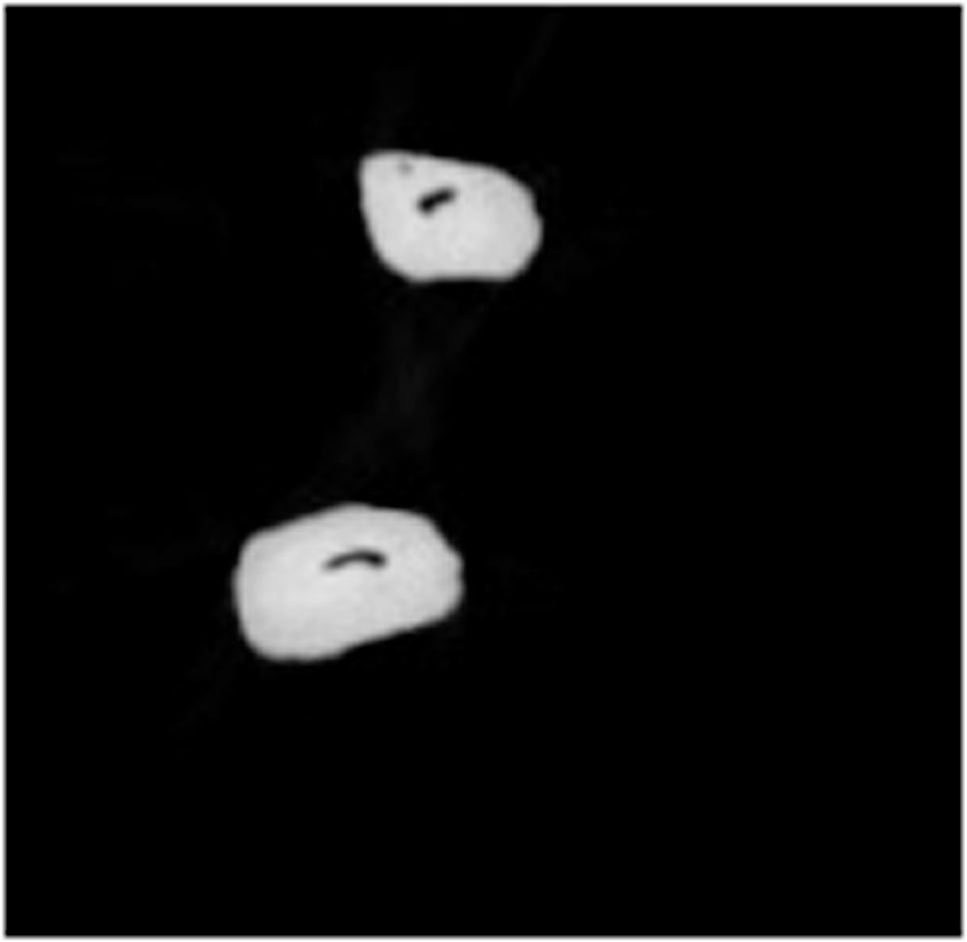
Single minor apical foramina observed in a hypomineralized tooth

Table 3 shows the number of minor apical foramen and major apical foramen. A statistically significant difference was observed between the hypomineralized and healthy groups in terms of the number of minor apical foramina in the middle third of the root [*p* = 0.016] and the total number of minor apical foramina. [*p* = 0.047] (Figs. 1 and 2).

**Table 3:** Statistical analysis showing the number of minor apical foramen and major apical foramen and the difference between the hypomineralized and healthy groups

Groups		Root number	Major apical foramen	Minor apical foramen			
				C	M	A	T
Hypomineralized teeth	Mean ± SD	1.80 ± 0.81	2.03 ± 0.93	0 ± 0	0.40 ± 0.67	3.57 ± 2.05	4.03 ± 2.16
Healthy teeth	Mean ± SD	1.73 ± 0.69	2.30 ± 1.15	0 ± 0	0.067 ± 0.25	2.83 ± 1.32	3.10 ± 1.27
	p value	0.732	0.327	-	0.016*	0.105	0.047*

Independent samples t tests were used for statistical analysis, with *p* < 0.05

*C *Cervical, *M *Middle, *A *Apical, *T *Total, *SD *Standard deviation.

* indicates a statistically significant result

**Fig. 3:**
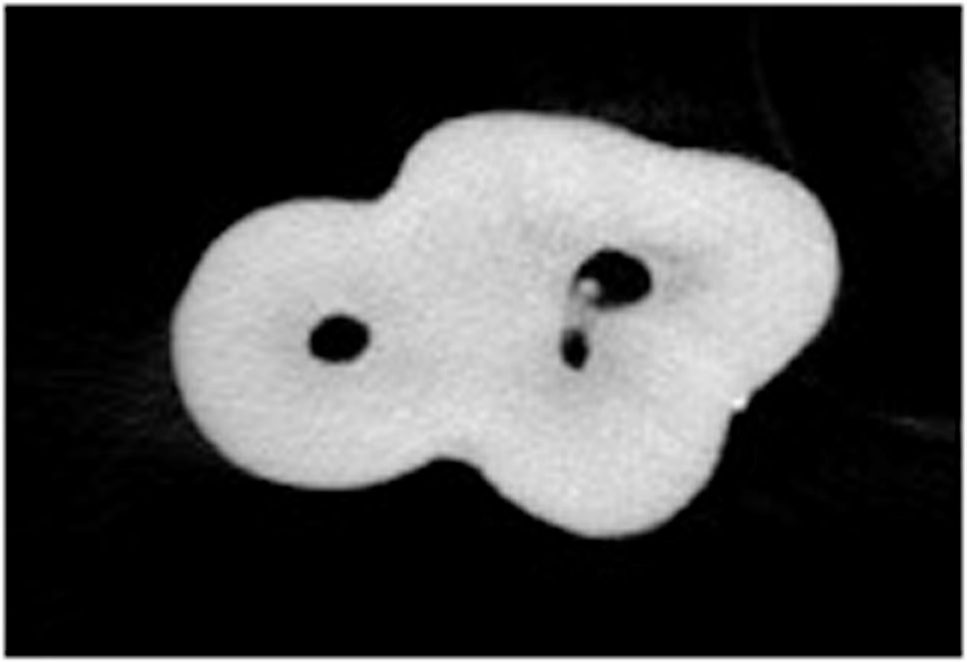
Isthmus observed in a healthy tooth

**Fig. 4:**
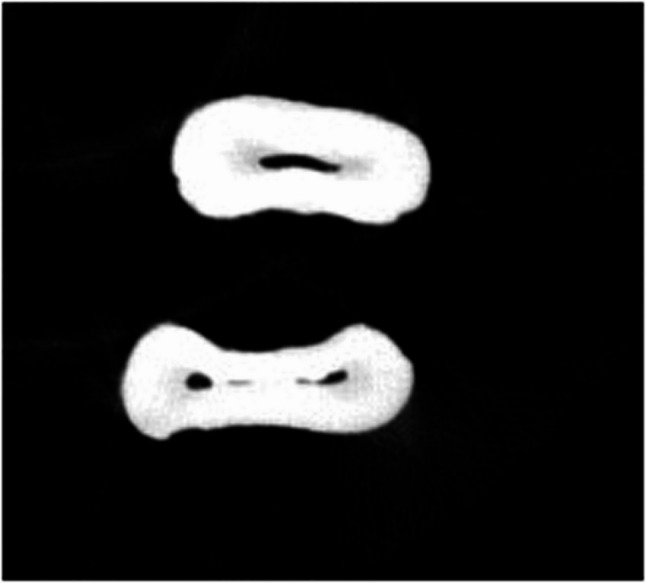
Isthmus observed in a hypomineralized tooth

Table 4 shows the total number of isthmuses per tooth. There was no statistically significant difference between the groups regarding the isthmus (Figs. 3 and 4).

**Table 4:** Statistical analysis showing the total number of isthmuses per tooth and the difference between the hypomineralized and healthy groups

Groups	Hypomineralization	Healthy	Hypomineralization	Healthy
Tooth number	Root number		Isthmus	
1	1	2	9	4
2	3	3	1	19
3	2	2	1	0
4	1	1	12	4
5	2	3	5	6
6	2	1	3	10
7	1	2	10	4
8	3	2	0	23
9	2	1	3	9
10	2	3	6	12
11	2	1	4	12
12	1	2	9	14
13	3	3	5	12
14	1	2	9	8
15	2	2	2	3
16	1	1	5	7
17	2	1	0	15
18	2	1	5	3
19	1	1	1	13
20	2	2	13	14
21	2	3	4	8
22	3	2	16	6
23	1	2	17	9
24	2	3	13	14
25	2	2	2	0
26	1	1	2	8
27	2	2	14	14
28	1	2	6	2
29	2	2	6	13
30	2	1	4	3
Mean ± SD	1.80 ± 0.67	1.87 ± 0.73	6.23 ± 4.88	8.97 ± 0.73
p value	0.71		0.49	

*SD *standard deviation

Independent samples t tests were used for statistical analysis, with *p* < 0.05

A statistically significant difference was observed between the two groups in terms of cervical width, with hypomineralized teeth exhibiting greater cervical width than healthy teeth. Table 5 shows the cervical thickness, cervical width and area measurements at the 1 mm, 5 mm, 7 mm, 9 mm and 11 mm levels from the apex hypomineralized and healthy groups. No statistically significant difference was observed between the two groups in terms of cervical thickness. Furthermore, there was no statistically significant difference between the groups in the root canal area measurements obtained at the 1, 3, 5, 7, 9, and 11 mm levels from the apex.

**Table 5:** Statistical analysis of cervical thickness, cervical width and area measurements at 1 mm, 3mm, 5 mm, 7 mm, 9 mm and 11 mm and the differences between the hypomineralized and healthy groups

Measurements		Groups		*p* value
		Hypomineralized	Healthy	
		Mean±SD	Mean±SD	
Cervical thickness		4.35±1.18	4.46±1.07	0.71
Cervical width		2.59±2.05	2.05±0.74	0.02*
Area	1 mm	0.42±0.39	0.26±0.22	0.06
	3 mm	0.53±0.65	0.43±0.8	0.42
	5 mm	0.71±0.45	0.61±0.49	0.43
	7 mm	0.66±0.74	0.63±0.71	0.86
	9 mm	0.64±1.14	0.92±1.63	0.45
	11 mm	0.41±0.95	0.20±0.74	0.33

Independent samples t tests were used for statistical analysis, with *p*<0.05

*SD *standard deviation, *mm *millimeter

* indicates a statistically significant result

## Discussion

Molar–incisor hypomineralization–affected teeth are clinically recognized to exhibit increased enamel porosity and reduced mechanical strength, conditions that facilitate rapid caries progression and predispose these teeth to early pulpal involvement [23, 24]. Understanding the internal morphological and structural characteristics of these teeth, such as their root canal configurations, major and minor foramina, and isthmus formations, may provide valuable insights for improving endodontic treatment outcomes. Although a limited number of studies have investigated root canal morphology in MIH-affected molars using different imaging modalities, including cone-beam computed tomography, detailed three-dimensional assessments based on high-resolution microcomputed tomography remain scarce [25, 26].

Various techniques have been utilized by researchers to examine the root canal morphology of teeth. These methods include endodontic access preparation followed by radiographic evaluation with files inserted into the canals [27], retrospective analysis of radiographic records [28], sectioning combined with macroscopic or scanning electron microscopic examination [29], and advanced imaging modalities such as cone-beam computed tomography [CBCT] [30], spiral computed tomography [29], and µCT [28]. Among these, µCT has been demonstrated to be a highly precise and nondestructive technique, allowing for detailed visualization and quantitative evaluation of root canal systems and their internal structures. The present study contributes to this gap by providing a detailed µCT evaluation of hypomineralized molars, offering a foundation for more predictable and conservative treatment approaches that may preserve these teeth rather than resorting to extraction.

The use of different methodologies has led to the development of various classification systems for assessing root canal configurations [15, 16, 18]. However, the main drawback of relying on a single classification system is that it may not be comprehensive enough to record all possible combinations of root canal configurations. Therefore, in the present study, the evaluation of root canal configurations was conducted via the classification proposed by Sert and Bayırlı [18], which includes a greater number of variations than previous systems do. In both the healthy and hypomineralized groups, the most frequently observed root canal configuration was Type 1 in the present study. Among the maxillary teeth, seven different configurations (Type 1,3,4,5,10,12,19) were identified in the healthy group, whereas four configurations (Type 1,2,3,15) were observed in the hypomineralized group. In the mandibular teeth, nine different configurations (Type 1,2,3,4,6,9,10,15,17) were detected in the healthy group, whereas seven canal configurations (Type 1,2,3,4,10,14,15) were observed in the hypomineralized group. Neboda [25] reported that in mandibular hypomineralized molar teeth, similar variations were noted compared with those in hypomineralized maxillary molars. In hypomineralized maxillary teeth, the greatest variation was observed in the mesiobuccal root, whereas in hypomineralized mandibular teeth, the mesial root exhibited more variation than did the distal root. In maxillary teeth, Type 1 was the most frequently observed configuration for all three roots. Among mandibular teeth, Type 1 was the most common configuration in single-rooted teeth, Type 4 was most frequently observed in the mesial roots, and Type 1 was the most prevalent configuration in the distal roots. Consistent with our findings, Somma [31] and Neboda [25] reported that in maxillary first permanent molars, the mesiobuccal canal exhibited greater variation than did the distal or palatal canals.

Reis et al. [26] reported that when hypomineralized and clinically healthy molars were evaluated, no statistically significant differences were found in the number of major and minor foramina. Similarly, in our study, no statistically significant difference was detected in the number of major foramina; however, unlike their findings, a significant difference in the number of minor apical foramina was detected, with hypomineralized teeth exhibiting a greater count. Briseño-Marroquín et al. [21] conducted a study in which major foramina were defined as those with a diameter of 0.25 mm or greater. Reis et al. [26] reported the presence of more than one major foramen in two roots of healthy mandibular teeth. In the present study, more than one major foramen was detected in 12 roots of healthy teeth and in 5 roots of hypomineralized teeth. These anatomical variations are of considerable clinical importance, as they may hinder precise and effective instrumentation, disinfection, and obturation of the root canal system, potentially leading to endodontic treatment failure.

In the present study, no statistically significant difference was found between healthy and hypomineralized teeth in terms of the number of isthmuses. When evaluated according to the type of isthmus, Type 1 was the most frequently observed configuration. In the cervical region, isthmuses were the least frequently observed area in both groups. Mehrvarzfar et al. [32] conducted a study and reported the highest prevalence of the isthmus at 6 mm from the apex [92%], whereas the lowest prevalence was observed at 2 mm from the apex [70%]. Previous studies have reported variable findings regarding isthmus characteristics in permanent molars, likely due to differences in evaluation methods, classification systems, and tooth types [15, 19, 28, 32, 33]. The present µCT -based study contributes to the literature by providing a three-dimensional, high-resolution comparison of isthmus features in hypomineralized and healthy molars, which may support endodontic treatment planning.

In the present study, no statistically significant differences were observed between hypomineralized and healthy teeth with respect to cervical thickness or dentin area measured at 1, 3, 5, 7, 9, and 11 mm from the root apex. Although the mean cervical width values tended to be higher in hypomineralized teeth. In contrast to our findings, Reis et al. [26] reported smaller linear measurements in hypomineralized teeth compared with healthy controls. These discrepancies may be attributed to difference imaging modality.

The hypersensitivity commonly associated with MIH has been attributed to increased enamel porosity and early exposure of dentinal tubules. In this context, the higher number of minor apical foramina and the increased cervical canal width observed in hypomineralized molars in the present study may be considered potential anatomical features facilitating pulpal irritation. Although no direct causal relationship can be inferred, these findings may contribute to the understanding of the increased endodontic challenges reported in MIH-affected teeth.

However, the present study has certain limitations. First, the sample size was limited due to the difficulty in obtaining hypomineralized molars, and the combined analysis of maxillary and mandibular molars may obscure tooth-specific differences. Second, the inclusion of both first and second molars may introduce additional anatomical variability; future studies may benefit from focusing solely on first molars to ensure consistency. Third, morphometric measurements were performed at fixed levels (1–11 mm) along the root, representing a two-dimensional approach that may not fully capture three-dimensional canal volume and shape variations. Future research with larger sample sizes, separate maxillary and mandibular evaluations, and enhanced 3D segmentation is warranted.

## Conclusion

This study indicates that hypomineralized teeth exhibit certain differences in root canal morphology when compared with healthy teeth. The observed morphological variations, particularly in specific root canal and hard tissue parameters, suggest that such differences should be taken into consideration during endodontic procedures in teeth affected by molar–incisor hypomineralization. An improved understanding of these anatomical features may contribute to more informed treatment planning and help anticipate potential clinical challenges. However, further clinical and experimental studies are required to clarify the extent to which these morphological differences influence endodontic outcomes.

## Data Availability

The author elects to not share data.The datasets used and/or analysed during the current study are available from the corresponding author on reasonable request.
